# Impaired Postural Control Reduces Sit-to-Stand-to-Sit Performance in Individuals with Chronic Obstructive Pulmonary Disease

**DOI:** 10.1371/journal.pone.0088247

**Published:** 2014-02-12

**Authors:** Lotte Janssens, Simon Brumagne, Alison K. McConnell, Kurt Claeys, Madelon Pijnenburg, Nina Goossens, Chris Burtin, Wim Janssens, Marc Decramer, Thierry Troosters

**Affiliations:** 1 KU Leuven Department of Rehabilitation Sciences, University of Leuven, Leuven, Belgium; 2 Centre for Sports Medicine and Human Performance, Brunel University, Uxbridge, United Kingdom; 3 KU Leuven Department of Rehabilitation Sciences, University of Leuven KULAB, Bruges, Belgium; 4 Respiratory Rehabilitation and Respiratory Division, University Hospital Leuven, Leuven, Belgium; 5 Department of Allied Health Professions, Fontys University of Applied Sciences, Eindhoven, Netherlands; Pulmonary Research Institute at LungClinic Grosshansdorf, Germany

## Abstract

**Background:**

Functional activities, such as the sit-to-stand-to-sit (STSTS) task, are often impaired in individuals with chronic obstructive pulmonary disease (COPD). The STSTS task places a high demand on the postural control system, which has been shown to be impaired in individuals with COPD. It remains unknown whether postural control deficits contribute to the decreased STSTS performance in individuals with COPD.

**Methods:**

Center of pressure displacement was determined in 18 individuals with COPD and 18 age/gender-matched controls during five consecutive STSTS movements with vision occluded. The total duration, as well as the duration of each sit, sit-to-stand, stand and stand-to-sit phase was recorded.

**Results:**

Individuals with COPD needed significantly more time to perform five consecutive STSTS movements compared to healthy controls (19±6 vs. 13±4 seconds, respectively; p = 0.001). The COPD group exhibited a significantly longer stand phase (p = 0.028) and stand-to-sit phase (p = 0.001) compared to the control group. In contrast, the duration of the sit phase (p = 0.766) and sit-to-stand phase (p = 0.999) was not different between groups.

**Conclusions:**

Compared to healthy individuals, individuals with COPD needed significantly more time to complete those phases of the STSTS task that require the greatest postural control. These findings support the proposition that suboptimal postural control is an important contributor to the decreased STSTS performance in individuals with COPD.

## Introduction

Functional limitations in individuals with chronic obstructive pulmonary disease (COPD) frequently restrict their performance of daily activities like walking, stair climbing and transport [1]. Recently, it has been shown that the functional limitations in individuals with COPD are not only attributed to their respiratory impairment, but also to the many extra-pulmonary consequences of the disease [2]. The extra-pulmonary mechanisms underlying the disability in individuals with COPD are not fully understood.

The ability to rise and sit down on a chair, usually called sit-to-stand-to-sit (STSTS), is an essential daily functional activity [3]. The STSTS performance can be limited by decreased muscle strength [4], but it also places a high demand on the postural balance system [5]. Moreover, it has been established that longer STSTS times in elderly strongly predict fall risk and functional dependence [6]–[8]. Interestingly, fall injuries [9], [10] and balance deficits [10]–[14] are higher in individuals with COPD compared to healthy individuals of similar age.

Recently, it has been shown that individuals with COPD are not able to achieve the same number of STSTS repetitions within one minute as healthy individuals [15], [16]. Moreover, STSTS seems strongly associated to mortality in COPD patients [17]. This functional limitation has been mainly attributed to their ventilatory restrictions and peripheral muscle weakness [15]. Moving from a sitting to a standing position, and the reverse, requires a coordinated vertical and horizontal displacement of the center of mass [18], [19]. However, the co-ordination of the STSTS movement has not been investigated in individuals with COPD. It is reasonable to hypothesize that the decreased STSTS performance in individuals with COPD may be at least partially attributable to suboptimal postural control. This can be examined by the analysis of distinct phases (sit, sit-to-stand, stand, stand-to-sit) of the STSTS movement based on center of pressure (CoP) trajectories [20], [21]. To our knowledge, possible postural control deficits underlying the STSTS performance in individuals with COPD have yet to be examined. We have previously speculated on the role of inadequate postural control in COPD in relation to impaired respiratory (diaphragm) muscle function [22].

Thus, the purpose of this study was to compare the performance and control of the STSTS task between individuals with COPD and healthy matched controls. We hypothesize that there will be impaired postural control during STSTS in individuals with COPD, which may be manifest in an increased time to stand up and sit down on a chair.

## Methods

### Subjects

Eighteen individuals with COPD (6 women, 12 men) and 18 healthy controls participated in this study. The groups were matched for age (+/−2 years) and gender. The individuals with COPD were recruited from a local sports group individualized for individuals with COPD. Individuals with a history of specific balance problems (i.e., diagnosed vestibular or neurological disorder), spinal surgery, or lower limb musculoskeletal problems (i.e., surgery, injury or pathology at hip, knee, ankle or foot) were excluded. All participants gave their written informed consent conform to the principles of the Declaration of Helsinki (1964). The study was approved by the local Ethics Committee of Biomedical Sciences, KU Leuven, Belgium (Clinical Trial Center: B322201112379-S53589) and registered at www.clinicaltrials.gov with identification number NCT01505543.

A physical activity questionnaire was completed [23]. Spirometry was evaluated using forced expiratory volume in one second (FEV1), forced vital capacity (FVC) and functional residual capacity (FRC). Respiratory muscle strength was evaluated by measuring maximal inspiratory pressure (PImax) and maximal expiratory pressure (PEmax) using an electronic pressure transducer (MicroRPM, Micromedical Ltd., Kent, UK). The PImax was measured at functional residual capacity and the PEmax at total lung capacity. A minimum of five repetitions were performed and tests were repeated until there was less than five % difference between the best and second best test. The highest pressure sustained over one second was recorded and compared to reference values [24]. Isometric hand grip force (HGF) was measured using a hydraulic hand grip dynamometer (Jamar Preston, Jackson, MI) [25]. Isometric quadriceps force (QF) was quantified in the individuals with COPD using a Cybex Norm Dynamometer (Cybex Norm, Enraf Nonius, Delft, The Netherlands). Peak extension torque was measured at 60° of knee flexion. At least three measurements were obtained and the highest reproducible value was taken into analysis and compared to reference values [26].

### Kinematics

Anterior-posterior CoP displacements were assessed using a six-channel force plate (Bertec, OH, USA). Force plate signals were sampled at 500 Hz using a Micro1401 data acquisition system using Spike2 software (Cambridge Electronic Design, UK) and were filtered using a low pass filter with a cut-off frequency of five Hz.

### Experimental protocol

The participants were instructed to sit barefoot on a stool on the force plate, with their arms relaxed along the body. The stool height was adjusted to create a 90 degree angle in both the hips and knees. The vision of the participants was occluded by means of non-transparent goggles, to minimize a potential effect of vision on the performance. After 15 seconds of usual sitting, the participants were asked to perform five STSTS movements, as fast as possible and with a full range of motion. An investigator was standing nearby the participant to prevent actual falls.

### Data reduction and statistical analysis

**Figure 1:**
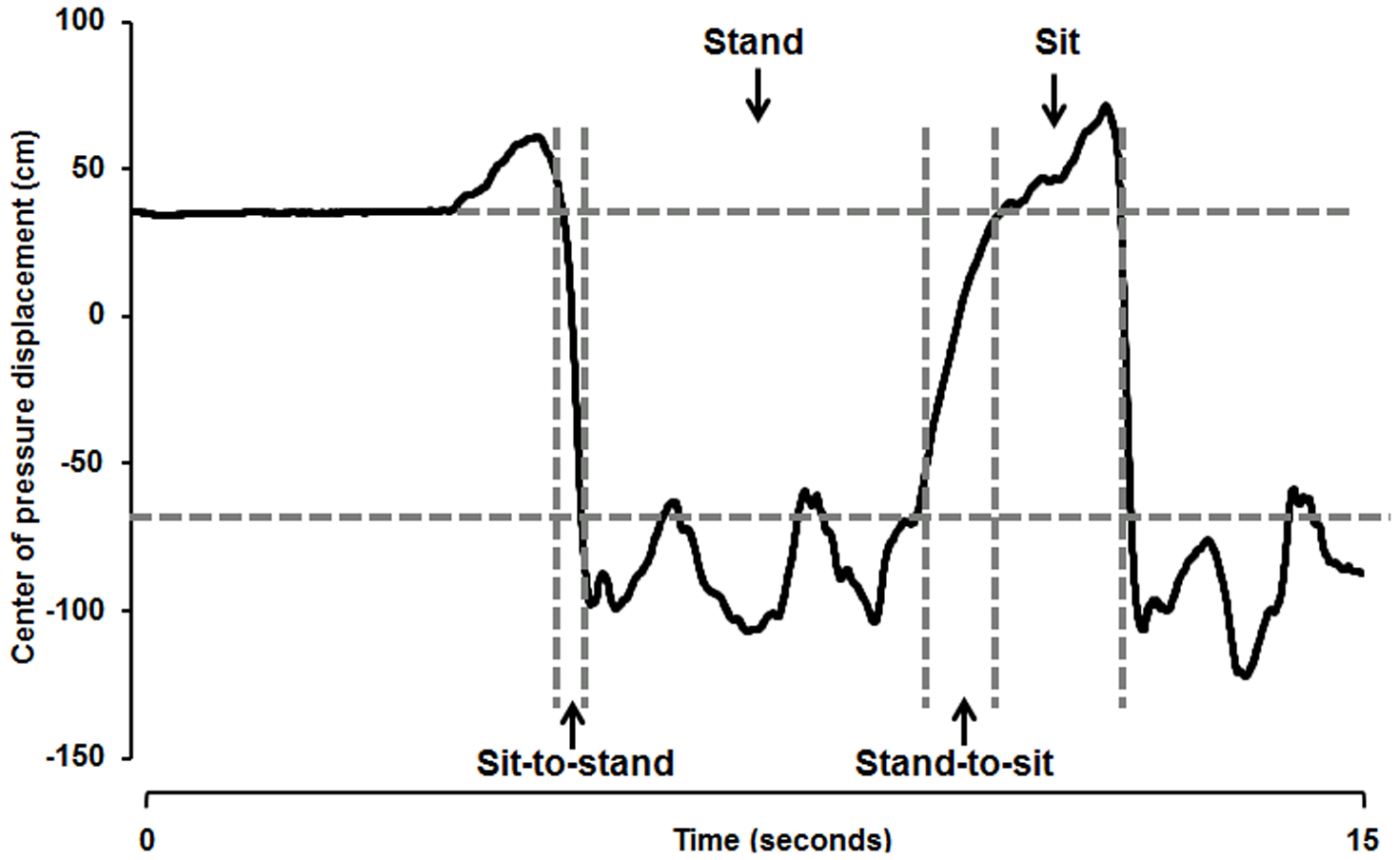
Raw data of center of pressure (CoP) displacement of a healthy individual during sit-to-stand-to-sit (STSTS). The different phases (sit-to-stand, stand, stand-to-sit, sit) were defined based on the mean value of CoP during usual sitting (upper horizontal grey line) and standing (lower horizontal grey line).

Force plate data were calculated using Spike2 software and Microsoft Excel. The total duration of the five consecutive STSTS movements, as well as the duration of each sit, sit-to-stand, stand and stand-to-sit phase was recorded. The subdivision in different phases was made based on the CoP displacements during the trial; to define the sit phase, the mean value of CoP during usual sitting was used; to define the stand phase, the mean value of CoP during usual standing was used (Figure 1) [20], [21].

A one-way analysis of variance (ANOVA) was used to examine differences in baseline characteristics between the two groups (Table 1). A repeated measures ANOVA was used to examine differences between subjects and within-subjects across the different STSTS phases. A Pearson coefficient was calculated to correlate between variables. A *post hoc* test (Tukey) was performed to further analyze these results in detail. The statistical analysis was performed with Statistica 9.0 (Statsoft, OK, USA) with the level of significance set at p<0.05.

**Table 1:** Participant characteristics.

	Control group	COPD group	p-value
**Age (yrs)**	64±7	65±7	0.926
**Height (cm)**	172±9	169±7	0.354
**Weight (kg)**	74±11	75±14	0.839
**BMI (kg/m^2^)**	25±3	26±4	0.361
**PAI**	9.1±1.7	8.2±1.1	0.114
**FVC (% pred)**	116±13	93±25	**0.002**
**FEV1 (% pred)**	109±16	51±19	**0.001**
**FEV1/FVC**	75±9	45±13	**0.001**
**FRC (% pred)**	N/A	146±36	N/A
**PImax (cmH_2_O)**	107±23	79±20	**0.001**
**PImax (% pred)**	113±24	85±23	**0.002**
**PEmax (cmH_2_O)**	192±48	152±37	**0.010**
**PEmax (% pred)**	119±25	99±31	**0.047**
**HGF (% pred)**	123±18	93±18	**0.002**
**QF (% pred)**	N/A	82±22	N/A

Data are presented as mean ± standard deviation. BMI: body mass index; PAI: physical activity index (maximum score = 15); FVC: forced vital capacity; FEV1: forced expiratory volume in 1 second; FRC: functional residual capacity; PImax: maximal inspiratory pressure; PEmax: maximal expiratory pressure; HGF: hand grip force; QF: quadriceps force; % pred: percentage predicted; Significant p-values (p<0.05) in bold.

## Results

### Participant characteristics

Table 1 displays the characteristics of both groups. The participants showed equivalent anthropometric characteristics and physical activity levels (p>0.05). The patients in the study had a known diagnosis of stable COPD based on the GOLD criteria (stages II-III) [27]. None of the healthy participants had a history of smoking or evidence of airflow obstruction. The individuals with COPD showed a significantly lower respiratory muscle strength compared to the healthy controls (p<0.05).

### Sit-to-stand-to-sit (STSTS) performance

The COPD group (19±6 seconds) required 46% more time to perform five consecutive STSTS movements with vision occluded, compared to the healthy group (13±4 seconds) (p = 0.001).

Within the COPD group, no correlation was found between the total time to perform five STSTS movements and the QF (% predicted) (r = −0.09, p = 0.770), or between the total STSTS time and the HGF (% predicted) (r = 0.268, p = 0.335), or between the total STSTS time and the inspiratory muscle strength (PImax (% predicted)) (r = −0.06, p = 0.804). However, when data from both groups were pooled, a significant negative correlation was observed between the total STSTS time and PImax (r = −0.35, p = 0.043).

### Sit-to-stand-to-sit (STSTS) phase duration

**Figure 2:**
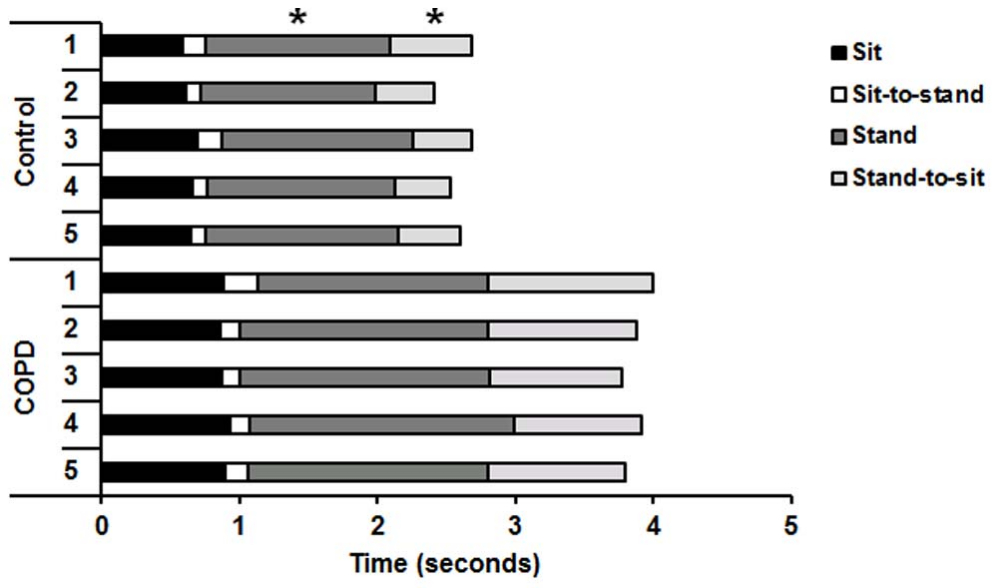
Mean durations of the five sit, sit-to-stand, stand and stand-to-sit phases. The phase durations of the sit-to-stand-to-sit (STSTS) task are displayed for the control group and COPD group. (* = p<0.05 between both groups for all five STSTS movements).

The longer total STSTS duration in the COPD group was explained primarily by a significantly longer stand phase (p = 0.028), and a significantly longer stand-to-sit phase (p = 0.001). The duration of the sit phase (p = 0.766), and the sit-to-stand phase (p = 0.999) did not differ between both groups. Figure 2 and Table 2 display the phase durations of the five STSTS movements in both groups. No correlation was found between the different phase durations and QF, HGF or PImax (p>0.05).

**Table 2:** Mean durations (in seconds) of the five sit, sit-to-stand, stand and stand-to-sit phases.

	Control group	COPD group	p-value
**Sit**	0.61±0.21	0.87±0.36	0.766
**Sit-to-stand**	0.11±0.12	0.14±0.08	0.999
**Stand**	1.27±0.39	1.79±0.78	**0.028**
**Stand-to-sit**	0.43±0.41	1.08±0.88	**0.001**

Data are presented as mean ± standard deviation. Significant p-values (p<0.05) in bold.

## Discussion

Individuals with COPD needed significantly more time to perform five consecutive STSTS movements, compared to healthy controls. This could be explained by longer stand and stand-to-sit phases, which are the phases requiring the greatest postural control, but not by any differences in the sit phases and the more muscle strength dependent sit-to-stand phases. These novel findings shed some light on a possible new and overlooked extra-pulmonary mechanism underlying the decreased functional capacity in individuals with COPD, more specifically impaired postural control.

Different underlying mechanisms may explain why individuals with COPD increase the time of the stand-to-sit phase (but not of the sit-to-stand phase) during STSTS. First, it has been shown that stand-to-sit requires more vertical control of the center of mass compared to sit-to-stand [18], especially in elderly [28]. The high requirement of fine trunk control may explain the longer stand-to-sit phase in the COPD group, suggesting a decreased ability to meet the normal postural control demand in this population. A second explanation for the longer stand-to-sit times observed in the COPD group may be found in the loss of trunk mobility induced by hyperinflation [29]. A free play of joints is essential to control balance in an effective way, especially when ventilator demand is increased [30]. Dubost et al. demonstrated a smaller trunk motion during stand-to-sit in elderly compared to young individuals, although no difference was found between groups in sit-to-stand performance [31]. Accordingly, it is reasonable to suggest that reduced spinal mobility in individuals with COPD places a higher demand on the postural control system during the stand-to-sit transfer and consequently increases the time needed to land on a chair. A third possible explanation is the need to decelerate and to land softly on a chair, which requires eccentric contraction of the quadriceps muscles. Accordingly, the longer stand-to-sit times may also be explained by a relatively greater demand upon the quadriceps muscles in individuals with COPD [32], caused by their quadriceps weakness (QF was 82% of the predicted value). Nonetheless, the contribution of QF in this study must be interpreted with care since QF was not measured in the control group and only isometric, in contrast to isokinetic QF was measured. Eccentric (vs. concentric) quadriceps contraction requires rather more muscle control than muscle force during sit-to-stand [33]. Butcher et al. report a strong association (r = 0.81) between eccentric QF and the STSTS performance in individuals with COPD, which may indicate that eccentric muscle control is a strong contributor to the daily function of individuals with COPD [34]. However, the current study did not identify an association between isometric QF and STSTS performance, but unfortunately, eccentric and isokinetic QF were not measured. Although it has been reported that resistance training may produce an increase in leg muscle strength, the effects on functional tasks such as STSTS is still in doubt [35]. Collectively, these observations support the hypothesis that muscle control and postural control may limit the STSTS performance, and other functional tasks, in individuals with COPD. Isokinetic QF measurements must further reveal whether the longer stand-to-sit phase can be additionally associated with quadriceps weakness.

In addition to longer stand-to-sit times, individuals with COPD showed a longer stand phase during consecutive STSTS movements. The standing position itself requires a specific postural control skill in order to decelerate and accelerate the body to change the movement direction. This transition phase requires additional control of the center of mass in order to prepare the body for the movement phase (i.e. stand-to-sit), and thus may last longer when postural control is impaired. Postural control depends on visual, vestibular and proprioceptive inputs [36]. More specifically, a suboptimal use of proprioception during postural control (i.e., decreased back proprioceptive use, increased ankle proprioceptive use) seems to predict this longer preparatory transition phase [20]. In this respect, the longer stand phases during the STSTS movements may be explained by the maladaptive proprioceptive changes in individuals with COPD, as it has been recently shown that these individuals adopt a suboptimal proprioceptive strategy during upright standing [22]; this may contribute to the observed balance deficits in individuals with COPD [10]–[14]. Furthermore, it is pertinent to mention that the diaphragm, a primary inspiratory muscle, has a major role in postural control [37]. Accordingly, postural control during STSTS might be compromised in individuals with COPD due to their impaired diaphragm function, as indicated by their reduced inspiratory muscle strength, as well as by the increased demand for trunk muscle contribution to breathing. Taken together, it may be hypothesized that the increased time individuals with COPD need to perform a set of STSTS movements, may not be due solely to reduced peripheral muscle strength as suggested before [4], but also to an impaired postural control. This hypothesis is supported by our observation that it were the most posturally challenging phases of the STSTS that were significantly extended in the COPD group.

Our findings may also help to explain why fall risk is highly associated to STSTS performance [6], [8], as many falls occur during activities involving a body transfer [38]. Additionally, fear of falling has often been reported in individuals with COPD [39], which might contribute to the increased stand and stand-to-sit times, since these are the most insecure phases of the STSTS task. Falls and fear of falling seem closely correlated in individuals with COPD [39]. Given the high prevalence of osteoporosis in individuals with COPD [40], an increased fall risk may lead to a significant loss of functionality and increase in healthcare costs.

Current training programs for individuals with COPD focus mainly on enhancing (concentric) peripheral muscle strength and aerobic capacity. However, the results of our study support the idea that specific postural control training should be considered in the rehabilitation of patients with COPD, in order to improve the performance of daily activities like STSTS, which may decrease the associated fall risk. Furthermore, postural control training might also improve movement efficiency during functional tasks, thereby reducing the ventilatory demand of patients who have a reduced ventilatory capacity.

Some limitations must be addressed. Despite similar scores on the physical activity questionnaire in both groups, we suggest future studies to record physical activity more objectively by validated monitors, since daily physical activity is generally reduced in individuals with COPD which may contribute to postural control [41]. Although the results of this study provide novel insights into the reduced STSTS performance in individuals with COPD, the lack of a more detailed kinematic analysis limits interpretation. Isokinetic QF recordings of both groups would provide insight into quadriceps weakness as a contributory factor to the longer stand-to-sit phase. Three-dimensional motion analysis may contribute to better understanding of pelvic control as there may be a delayed and decreased pelvic movement during the STSTS transfer [20], contributing to the suboptimal postural control. Furthermore, prospective studies are required to shed light on whether the longer stand and stand-to-sit phases predict the fall incidence in individuals with COPD. The potential contribution of inspiratory muscle function to STSTS performance raises the intriguing question of whether specific training of these muscles might improve postural control; this intervention has already been shown to impart a number of other functional benefits to individuals with COPD [42].

In conclusion, individuals with COPD needed significantly more time to perform a series of STSTS movements compared to healthy controls. Interestingly, this could be explained by longer stand and stand-to-sit phases, but not by longer sit and sit-to-stand phases. The affected STSTS phases require the greatest postural control, and the results of this study therefore provide evidence that suboptimal postural control may contribute to the decreased STSTS performance in individuals with COPD. Our study encourages specific interventions addressing these mechanisms to improve the daily function in individuals with COPD.

## References

[1] Leidy NK (1995) Functional performance in people with chronic obstructive pulmonary disease. Image J Nurs Sch 27: 23–34. 7721307 10.1111/j.1547-5069.1995.tb00809.x

[2] Eisner MD, Iribarren C, Blanc PD, Yelin EH, Ackerson L, et al (2011) Development of disability in chronic obstructive pulmonary disease: beyond lung function. Thorax 66: 108–114. 21047868 10.1136/thx.2010.137661 PMC3111223

[3] Dall PM, Kerr A (2010) Frequency of the sit to stand task: An observational study of free-living adults. Appl Ergon 41: 58–61. 19450792 10.1016/j.apergo.2009.04.005

[4] Hughes MA, Myers BS, Schenkman ML (1996) The role of strength in rising from a chair in the functionally impaired elderly. J Biomech 29: 1509–1513. 8945648

[5] Lord SR, Murray SM, Chapman K, Munro B, Tiedemann A (2002) Sit-to-stand performance depends on sensation, speed, balance, and psychological status in addition to strength in older people. J Gerontol A Biol Sci Med Sci 57: M539–43. 12145369 10.1093/gerona/57.8.m539

[6] Buatois S, Miljkovic D, Manckoundia P, Gueguen R, Miget P, et al (2008) Five times sit to stand test is a predictor of recurrent falls in healthy community-living subjects aged 65 and older. J Am Geriatr Soc 56: 1575–1577. 18808608 10.1111/j.1532-5415.2008.01777.x

[7] Whitney SL, Wrisley DM, Marchetti GF, Gee MA, Redfern MS, Furman JM (2005) Clinical measurement of sit-to-stand performance in people with balance disorders: validity of data for the Five-Times-Sit-to-Stand Test. Phys Ther 85: 1034–1045. 16180952

[8] Najafi B, Aminian K, Loew F, Blanc Y, Robert PA (2002) Measurement of stand-sit and sit-stand transitions using a miniature gyroscope and its application in fall risk evaluation in the elderly. IEEE Trans Biomed Eng 49: 843–851. 12148823 10.1109/TBME.2002.800763

[9] Lawlor DA, Patel R, Ebrahim S (2003) Association between falls in elderly women and chronic diseases and drug use: cross sectional study. BMJ 327: 712–717. 14512478 10.1136/bmj.327.7417.712 PMC200802

[10] Roig M, Eng JJ, MacIntyre DL, Road JD, FitzGerald JM, et al (2011) Falls in people with chronic obstructive pulmonary disease: an observational cohort study. Respir Med 105: 461–469. 20869227 10.1016/j.rmed.2010.08.015 PMC3350813

[11] Beauchamp MK, Sibley KM, Lakhani B, Romano J, Mathur S, et al (2012) Impairments in Systems Underlying Control of Balance in COPD. Chest 141: 1496–1503. 22116798 10.1378/chest.11-1708

[12] Butcher SJ, Meshke JM, Sheppard MS (2004) Reductions in functional balance, coordination, and mobility measures among patients with stable chronic obstructive pulmonary disease. J Cardiopulm Rehabil 24: 274–280. 15286536 10.1097/00008483-200407000-00013

[13] Chang AT, Seale H, Walsh J, Brauer SG (2008) Static balance is affected following an exercise task in chronic obstructive pulmonary disease. J Cardiopulm Rehabil Prev 28: 142–145. 18360191 10.1097/01.HCR.0000314209.17300.cc

[14] Smith MD, Chang AT, Seale HE, Walsh JR, Hodges PW (2010) Balance is impaired in people with chronic obstructive pulmonary disease. Gait Posture 31: 456–460. 20206529 10.1016/j.gaitpost.2010.01.022

[15] Ozalevli S, Ozden A, Itil O, Akkoclu A (2007) Comparison of the Sit-to-Stand Test with 6 min walk test in patients with chronic obstructive pulmonary disease. Respir Med 101: 286–293. 16806873 10.1016/j.rmed.2006.05.007

[16] Rocco CC, Sampaio LM, Stirbulov R, Corrêa JC (2011) Neurophysiological aspects and their relationship to clinical and functional impairment in patients with chronic obstructive pulmonary disease. Clinics 66: 125–129. 21437448 10.1590/S1807-59322011000100022 PMC3044581

[17] Puhan MA, Siebeling L, Zoller M, Muggensturm P, Ter Riet G (2013) Simple functional performance tests and mortality in COPD. Eur Respir J 42: 956–963. 23520321 10.1183/09031936.00131612 PMC3787814

[18] Reisman DS, Scholz JP, Schöner G (2002) Coordination underlying the control of whole body momentum during sit-to-stand. Gait Posture 15: 45–55. 11809580 10.1016/s0966-6362(01)00158-8

[19] Kralj A, Jaeger RJ, Munih M (1990) Analysis of standing up and sitting down in humans: definitions and normative data presentation. J Biomech 23: 1123–1138. 2277047 10.1016/0021-9290(90)90005-n

[20] Claeys K, Dankaerts W, Janssens L, Brumagne S (2012) Altered preparatory pelvic control during the sit-to-stance-to-sit movement in people with non-specific low back pain. J Electromyogr Kinesiol 22: 821–828. 22595702 10.1016/j.jelekin.2012.04.007

[21] Arcelus A, Herry CL, Goubran RA, Knoefel F, Sveistrup H, Bilodeau M (2009) Determination of sit-to-stand transfer duration using bed and floor pressure sequences. IEEE Trans Biomed Eng 56: 2485–2492. 19622434 10.1109/TBME.2009.2026733

[22] Janssens L, Brumagne S, McConnell AK, Claeys K, Pijnenburg M, et al (2013) Proprioceptive changes impair balance control in individuals with chronic obstructive pulmonary disease. PLoS One 8: e57949. 23469255 10.1371/journal.pone.0057949 PMC3585868

[23] Baecke JA, Burema J, Frijters JE (1982) A short questionnaire for the measurement of habitual physical activity in epidemiological studies. Am J Clin Nutr 36: 936–942. 7137077 10.1093/ajcn/36.5.936

[24] Rochester DF, Arora NS (1983) Respiratory muscle failure. Med Clin North Am 67: 573–597. 6341727 10.1016/s0025-7125(16)31190-7

[25] Mathiowetz V, Kashman N, Volland G, Weber K, Dowe M, Rogers S (1985) Grip and pinch strength: normative data for adults. Arch Phys Med Rehabil 66: 69–74. 3970660

[26] Decramer M, Lacquet LM, Fagard R, Rogiers P (1994) Corticosteroids contribute to muscle weakness in chronic airflow obstruction. Am J Respir Crit Care Med 150: 11–16. 8025735 10.1164/ajrccm.150.1.8025735

[27] Global strategy for diagnosis, management, and prevention of COPD. Available: http://www.goldcopd.com. Accessed 01 September 2013.

[28] Mourey F, Pozzo T, Rouhier-Marcer I, Didier JP (1998) A kinematic comparison between elderly and young subjects standing up from and sitting down in a chair. Age Ageing 27: 137–146. 16296673 10.1093/ageing/27.2.137

[29] Engel R, Vemulpad S (2011) The role of spinal manipulation, soft-tissue therapy, and exercise in chronic obstructive pulmonary disease: a review of the literature and proposal of an anatomical explanation. J Altern Complement Med 17: 797–801. 21838523 10.1089/acm.2010.0517

[30] Kuznetsov NA, Riley MA (2012) Effects of breathing on multijoint control of center of mass position during upright stance. J Mot Behav 44: 241–253. 22671566 10.1080/00222895.2012.688894

[31] Dubost V, Beauchet O, Manckoundia P, Herrmann F, Mourey F (2005) Decreased trunk angular displacement during sitting down: an early feature of aging. Phys Ther 85: 404–412. 15842189

[32] Janaudis-Ferreira T, Wadell K, Sundelin G, Lindström B (2006) Thigh muscle strength and endurance in patients with COPD compared with healthy controls. Respir Med 100: 1451–1457. 16337114 10.1016/j.rmed.2005.11.001

[33] Duchateau J, Baudry S (2013) Insights into the neural control of eccentric contractions. J Appl Physiol 2013 Feb 21 [Epub ahead of print]. 10.1152/japplphysiol.00002.2013 23429873

[34] Butcher SJ, Pikaluk BJ, Chura RL, Walkner MJ, Farthing JP, Marciniuk DD (2012) Associations between isokinetic muscle strength, high-level functional performance, and physiological parameters in patients with chronic obstructive pulmonary disease. Int J Chron Obstruct Pulmon Dis 7: 537–542. 22973094 10.2147/COPD.S34170 PMC3430119

[35] O'Shea SD, Taylor NF, Paratz JD (2009) Progressive resistance exercise improves muscle strength and may improve elements of performance of daily activities for people with COPD: a systematic review. Chest 136: 1269–1283. 19734323 10.1378/chest.09-0029

[36] Lackner JR, DiZio P (2005) Vestibular, proprioceptive, and haptic contributions to spatial orientation. Annu Rev Pshychol 56: 115–147. 10.1146/annurev.psych.55.090902.142023 15709931

[37] Hodges PW, Gandevia SC (2000) Activation of the human diaphragm during a repetitive postural task. J Physiol 522: 165–175. 10618161 10.1111/j.1469-7793.2000.t01-1-00165.xm PMC2269747

[38] Nyberg L, Gustafson Y (1995) Patient falls in stroke rehabilitation. A challenge to rehabilitation strategies. Stroke 26: 838–842. 7740577 10.1161/01.str.26.5.838

[39] Hellström K, Vahlberg B, Urell C, Emtner M (2009) Fear of falling, fall-related self-efficacy, anxiety and depression in individuals with chronic obstructive pulmonary disease. Clin Rehabil 23: 1136–1144. 19906765 10.1177/0269215509342329

[40] Lehouck A, Boonen S, Decramer M, Janssens W (2011) COPD, bone metabolism, and osteoporosis. Chest 139: 648–657. 21362651 10.1378/chest.10-1427

[41] Troosters T, Sciurba F, Battaglia S, Langer D, Valluri SR, et al (2010) Physical inactivity in patients with COPD, a controlled multi-center pilot-study. Respir Med 104: 1005–1011. 20167463 10.1016/j.rmed.2010.01.012 PMC3471783

[42] Gosselink R, De Vos J, van den Heuvel S, Segers J, Decramer M, Kwakkel G (2011) Impact of inspiratory muscle training in patients with COPD: what is the evidence? Eur Respir J 37: 416–425. 21282809 10.1183/09031936.00031810

